# Associations of Genetic Polymorphisms of mTOR rs2295080 T/G and rs1883965 G/A with Susceptibility of Urinary System Cancers

**DOI:** 10.1155/2022/1720851

**Published:** 2022-01-17

**Authors:** Zhichao Min, Yuanyuan Mi, Zhiwei Lv, Yangyang Sun, Bowen Tang, Hao Wu, Ze Zhang, Hong Pan, Yujuan Zhang, Chao Lu, Li Zuo, Lifeng Zhang

**Affiliations:** ^1^Department of Urology, The First People's Hospital of Hangzhou Lin'an District, Hangzhou 310000, China; ^2^Department of Urology, Affiliated Hospital of Jiangnan University, Wuxi 214000, China; ^3^Department of Urology, The Affiliated Changzhou No. 2 People's Hospital of Nanjing Medical University, 29 Xinglong Road, Changzhou 213003, China; ^4^Department of Pathology, The Affiliated Changzhou No. 2 People's Hospital of Nanjing Medical University, 29 Xinglong Road, Changzhou 213003, China; ^5^Department of Operation Theatre, The Affiliated Changzhou No. 2 People's Hospital of Nanjing Medical University, 29 Xinglong Road, Changzhou 213003, China

## Abstract

**Background:**

Genetic polymorphisms in *mammalian target of rapamycin* (mTOR) signaling axis can influence the susceptibility of cancer. The relationship between mTOR gene variants rs2295080 T/G and rs1883965 G/A and the risk of cancer remains inconsistent. The present study is aimed at comprehensively investigating the association between mTOR polymorphisms and susceptibility to cancer.

**Methods:**

We conducted a comprehensive assessment using odds ratios (ORs), corresponding 95% confidence intervals (CIs), and in silico tools to evaluate the effect of mTOR variations. Immunohistochemical staining (IHS) and GSEA analysis were used to investigate the expression of mTOR in urinary system cancer.

**Results:**

The pooled analysis involved 22 case-control studies including 14,747 cancer patients and 16,399 controls. The rs2295080 T/G polymorphism was associated with the risk of cancer (G-allele versus T-allele, OR = 0.89, 95%CI = 0.80–0.98, *P* = 0.023; GT versus TT, OR = 0.88, 95%CI = 0.81–0.96, *P* = 0.004; GG+GT versus TT, OR = 0.87, 95%CI = 0.78–0.96, *P* = 0.008), especially for cancers of the urinary system, breast, and blood. Variation rs1883965 G/A was associated with cancer susceptibility, especially for digestive cancer. IHS analysis showed that mTOR was upregulated in prostate and bladder cancer. GSEA revealed that the insulin signaling pathway, lysine degradation pathway, and mTOR signaling pathway were enriched in the high mTOR expression group.

**Conclusions:**

The mTOR rs2295080 T/G polymorphism may be associated with susceptibility of urinary cancer. The expression of mTOR is positively correlated with tumor malignancy in prostate cancer.

## 1. Introduction

Malignant tumors are a major global public health problem [1]. Over the past decades, cancer-related morbidity and mortality have increased. In 2015, there were approximately 4.2 million new cancer cases in China and 2.8 million cancer-related deaths [2]. By 2018, the number of new carcinoma cases was expected to exceed 4.3 million, with 2.9 million deaths due to carcinoma [3]. Tumors arise due to the interaction of multiple environmental and internal factors. The internal factors are mainly manifested as changes of immune status and endocrine disorders and genetic mutations of vital signal transduction pathways [4, 5]. In *Homo sapiens*, missing phosphatase and tensin homologs of the phosphoinositide 3-kinase (PI3K)/AKT/mTOR signal transduction pathway on chromosome 10 are often activated in various carcinomas. They are involved in different cellular processes that include cell proliferation, angiogenesis, and tumorigenesis [6–8]. Mutation of central gene in the mTOR pathway could affect protein transcription and change the capacity of this pathway, which may be vital in carcinogenesis [9–11].

The mTOR gene, also known as FKBP12 rapamycin-associated protein (FRAP), acts as an essential serine-threonine kinase in signal transduction and participates in the biological processes of cell cycle, survival, and autophagy [12–14]. The human mTOR gene comprises 59 exons and is located on chromosome 1p36.2 [15, 16]. The gene is approximately 156 kb in length and plays a central role in the PI3K/AKT/mTOR pathway [17, 18]. The aberrant regulation of the mTOR signal transduction pathway has been implicated in a wide spectrum of carcinomas [19, 20]. Abnormal expression of mTOR has been described in several types of cancers, including kidney renal clear cell carcinoma (KIRC), bladder cancer (BLCA), primary liver cancer, esophageal carcinoma, and colorectal adenocarcinoma [21–25]. The changes may be associated with single-nucleotide polymorphisms (SNPs) and genetic mutations in the human genome [26, 27].

Previous studies have evaluated the association of genetic variants of the mTOR gene with the susceptibility to cancer. Results of a meta-analysis that included five eligible studies indicated that the rs2295080 TT genotype was related to increased cancer risk, but not with poor clinical outcome [28]. Another meta-analysis based on nine studies published several years later revealed that the rs2295080 T/G variant was associated with an increased risk of leukemia and a decreased risk of genitourinary cancers [29]. The correlation between variants of rs2295080 T/G or rs1883965 G/A and cancer risk remain unclear [30–33].

The current study sought to identify all eligible case-control studies to comprehensively assess the association between mTOR variants rs2295080 T/G or rs1883965 G/A and susceptibility to cancer [30–49]. *In silico* tools and immunohistochemical staining (IHS) analysis were used to investigate the expression of mTOR in three main urinary system cancers.

## 2. Materials and Methods

### 2.1. Search Strategy

The PMC, Embase, Chinese National Knowledge Infrastructure, and Google Scholar databases were searched for potentially relevant published studies. The search terms were (“rs2295080” OR “rs1883965” OR “mTOR” OR “Mammalian target of rapamycin”) AND (“cancer” OR “tumor” OR ”carcinoma”) AND (“mutant” OR “variant” OR “variation”). The most recent search update was 01 March 2021. In addition to the databases, we also screened qualified research by checking references from published articles.

### 2.2. Inclusion and Exclusion Criteria

Studies meeting the following criteria were included: (a) case-control or cohort studies addressing the association between the variation mTOR rs2295080 T/G or rs1883965 G/A and risk of cancer, (b) sufficient data of genotype frequencies to evaluate ORs and 95% CIs, and (c) articles written in English or other languages. The major exclusion criteria were as follows: (a) lack of a control group, (b) insufficient data to calculate ORs, and (c) no relevance to mTOR rs2295080 T/G or rs1883965 G/A variants and cancer risk.

### 2.3. Data Extraction

Study characteristics retrieved from eligible studies included the surname of the first author, publication date, origin of participants, type of cancer, source of control, ethnicity, gene distribution of mTOR polymorphisms, *P* value of Hardy-Weinberg equilibrium (HWE) in controls, and method of genotyping. In the stratification analysis, acute lymphoblastic leukemia and acute myeloid leukemia were classified as blood cancer. Urinary system tumors include BLCA, PRAD, and KIRC. Carcinomas of the digestive system included esophageal squamous cell carcinoma, gastric cancer, and colorectal cancer. The study included Asian populations and was divided into West Asian and East Asian groups.

### 2.4. Statistical Analyses

ORs and 95% CIs were used to evaluate the strength of association between the mTOR variants rs2295080 T/G or rs1883965 G/A and cancer susceptibility. For the rs2295080 T/G variation, allelic comparison refers to G-allele versus (vs.) T-allele. The heterozygous, homozygous, dominant, and recessive models represent GT vs. TT, GG vs. TT, GG+GT vs. TT, and GG vs. GT+TT. For the rs1883965 G/A variation, these five genetic models were A-allele vs. G-allele, AG vs. GG, AA vs. GG, AA+AG vs. GG, and AA and AG+GG. Heterogeneity of studies was investigated by the *Q* statistic test. *P* < 0.05 indicated statistical significance. A *P* value of heterogeneity < 0.05 indicated heterogeneity among the studies. A random effects model (DerSimonian and Laird) was conducted to calculate the ORs. Otherwise, a fixed effects model (Mantel–Haenszel) was performed. A *P* value of HWE was evaluated by Fisher's exact test. Subgroup analyses included the type of cancer, ethnicity, source of control, and genotype method. Begg's funnel plot was adopted to evaluate publication bias. The reliability of the included case-control studies was assessed by sensitivity analysis. STATA 11.0 software (StataCorp, College Station, TX, USA) was used for all statistical analyses.

### 2.5. In Silico and IHS Analysis of mTOR

We utilized an online database to investigate the MAFs in global and subpopulations (https://www.ncbi.nlm.nih.gov/snp). The expression of mTOR in human tissues was evaluated by another database (http://gemini.cancer-pku.cn/). Gene expression profiles of mTOR across various types of cancers and paired normal tissues were also assessed. We also employed online databases to detect the expression of mTOR in three main urinary system cancer, gene-gene connection, and overall survival time (http://ualcan.path.uab.edu/analysis.html; http://gepia.cancer-pku.cn/index.html). We also used TCGA database and GEPIA databases to explore the expression of mTOR in PRAD based on different molecular signature. STRING tools were used to assess the protein-protein crosstalk of mTOR (https://string-db.org/cgi/input.pl). We conducted IHS analyses to investigate mTOR expression of PRAD participants enrolled in our institute according to general standards [50]. Hematoxylin and eosin staining were used to confirm carcinoma in paraffin-embedded samples. Xylene was used to dewax tissue sections and alcohol was utilized to dehydrate the sections. Sections were washed twice using phosphate-buffered saline. A monoclonal antibody against mTOR was utilized (1 : 200 dilution, Abcam). The evaluation of IHS staining was performed by two experienced pathologists using a unified standard and a single blind method. The expression of mTOR was evaluated using a score ranging from 1 to 9. This study was approved by the Ethics Committee of the Affiliated Hospital of Jiangnan University, the First People's Hospital of Hangzhou Lin'an District, and the Affiliated Changzhou No. 2 People's Hospital of Nanjing Medical University. Furthermore, we used GSEA to investigate the potential activation signaling pathways of high mTOR expression group. The annotated gene set, c2.cp.kegg.v7.1.symbols.gmt, was selected as the reference gene set [51].

## 3. Results

### 3.1. Characteristics of Eligible Studies

**Figure 1:**
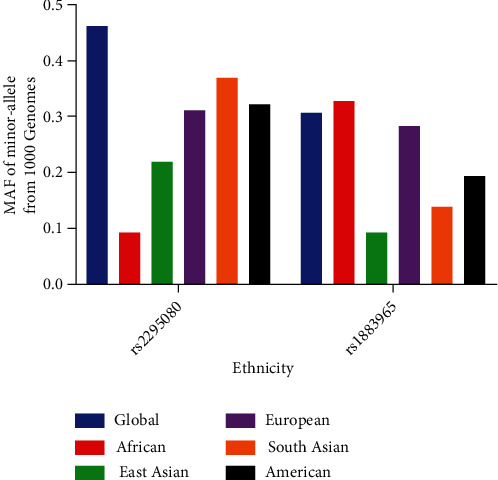
Minor allele frequencies of mTOR rs2295080 T/G and rs1883965 G/A variants in various races.

In total, 22 case-control studies comprising 14,747 cancer patients and 16,399 controls were included in the pooled analysis (Table 1). For the rs2295080 T/G variation, 18 studies with 10, 447 cancer patients and 11, 979 control subjects were analyzed. Stratified analysis by cancer type included six studies on urinary system cancer, six studies on digestive cancer, three studies on blood cancer, two studies on breast cancer, and one study on other cancer (thyroid cancer). In subgroup analysis by control source, 17 studies were hospital-based and the remaining six studies were population-based. In stratified analysis by ethnicity, 17 studies focused on East Asians and one on West Asians. In subgroup analysis by a genotype method, 14 studies utilized TaqMan assay. Three studies used polymerase chain reaction-restriction fragment length polymorphism (PCR-RFLP), and one study performed Sequenom MassARRAY. For the rs1883965 G/A variation, four studies with 4,300 cancer patients and 4,420 controls were included. Three studies involved digestive cancer and one involved urinary cancer. Subgroup analysis by control source included three population-based studies and one hospital-based study. All these studies involved East Asian populations. The classic genotyping method, TaqMan assay, was adopted by all these studies. We investigated the minor allele frequencies (MAFs) of mTOR rs2295080 and rs1883965 polymorphisms in various races. The MAFs for the rs2295080 variant were as follows: Americans, 0.320; Africans, 0.093; global population, 0.462; East Asians, 0.217; Europeans, 0.311; and South Asians, 0.370. The MAFs for rs1883965 were as follows: Americans, 0.195; Africans, 0.330; global population, 0.306; East Asians, 0.091; Europeans, 0.284; and South Asians, 0.138 (Figure 1).

### 3.2. Overall and Stratified Analyses

**Figure 2:**
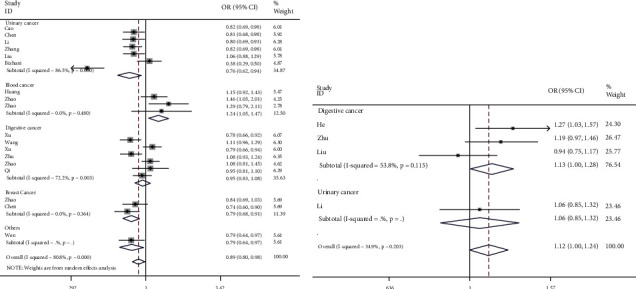
Forest plot of ORs for the relationship between mTOR variants rs2295080 T/G (a) or rs1883965 G/A (b) and risk of cancer (heterozygous comparison, random effects) in stratification analysis by type of cancer.

The strength of the association between mTOR variation rs2295080 T/G or rs1883965 G/A and susceptibility of cancer is shown in Table 2. Significant correlation with the likelihood of cancer was evident for SNP rs2295080 in the pooled data. Compared with individuals with T-allele, individuals with G-allele had an 11% lower risk of cancer (95%CI = 0.80-0.98, *P* = 0.023, Figure 2(a)). In subgroup analysis by cancer type, individuals carrying the G-allele had a 24% lower risk of urinary cancer, compared with those carrying the T-allele (95%CI = 0.62-0.94, *P* = 0.010). Similar findings were observed for the heterozygous contrast (95%CI = 0.70-0.85, *P* < 0.001) and dominant models (95%CI = 0.62-0.88, *P* = 0.001). For breast cancer, individuals with the G-allele had a 21% lower risk of cancer (95%CI = 0.68-0.91, *P* = 0.001). Similar results were indicated for homozygous contrast (95%CI = 0.27-0.66, *P* < 0.001), dominant model (95%CI = 0.68-0.95, *P* = 0.012), and recessive model (95%CI = 0.29-0.71, *P* < 0.001). Individuals with the G-allele had a 1.24-fold higher risk of blood cancer (95%CI = 1.05-1.47, *P* = 0.013). Similar results were found in the homozygous contrast (95%CI = 1.36-3.30, *P* = 0.001) and recessive model (95%CI = 1.34-3.22, *P* = 0.001). In stratified analysis by ethnicity, East Asians carrying the G-allele had an 8% lower risk of cancer than those with the T-allele (95%CI = 0.85-1.00, *P* = 0.044). Similar findings were indicated in population-based studies and using a TaqMan assay method. For the rs1883965 G/A variation, individuals carrying A-allele had a 1.12-fold higher likelihood of cancer than those carrying the G-allele (95%CI = 1.00-1.24, *P* = 0.045) (Figure 2(b)). In subgroup analysis by cancer type, a significant correlation with the susceptibility of digestive cancer was evident in the allelic contrast (95%CI = 1.00-1.28, *P* = 0.044), heterozygous (95%CI = 1.03-1.34, *P* = 0.014), and dominant models (95%CI = 1.02-1.32, *P* = 0.022). Similar results were confirmed in the population-based studies (allelic contrast, 95%CI = 1.04-1.33, *P* = 0.009; heterozygous comparison, 95%CI = 1.04-1.36, *P* = 0.011; and dominant model, 95%CI = 1.05-1.36, *P* = 0.009).

### 3.3. In Silico and IHC Analyses of mTOR

**Figure 3:**
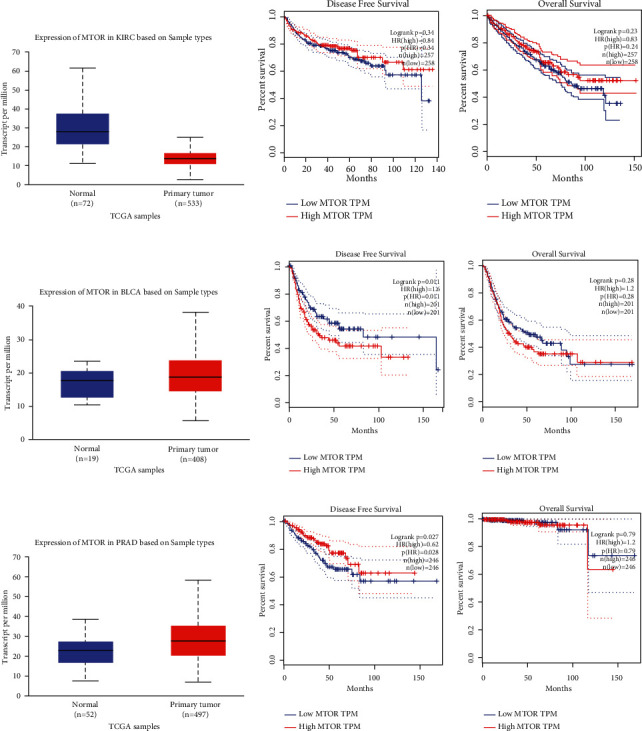
*In silico* analysis of mTOR expression based on sample types. The mTOR expression in kidney renal clear cell carcinoma (KIRC) was shown in (a). Effect of mTOR level on KIRC participants' overall survival (OS) and disease-free survival (DFS) time is shown in (b) and (c). The expression of mTOR in bladder cancer (BLCA) is shown in (d). Effect of mTOR level on BLCA patients' OS and DFS time is shown in (e) and (f). Expression of mTOR in prostate cancer (PRAD) is described in (g). PRAD patients with low expression of mTOR may have shorter disease-free survival (DFS) time than high mTOR expression group (h) (*P* < 0.05). No obvious difference was indicated on OS time (i) (*P* < 0.05).

**Figure 4:**
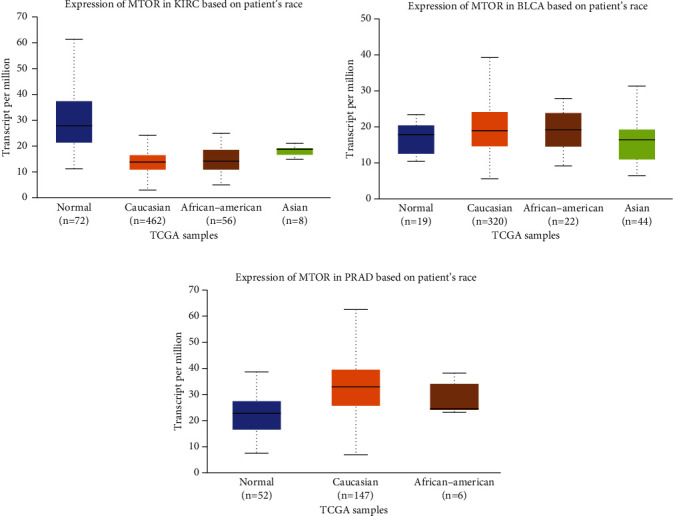
Expression of mTOR in urinary cancer based on patients' race. The expression of mTOR is downregulated in KIRC with Caucasian, African-American, and Asian descendants (a). Expression of mTOR is upregulated in BLCA with Caucasian and African-American descendants (b). Expression of mTOR is upregulated in Caucasian PRAD patients (c).

**Figure 5:**
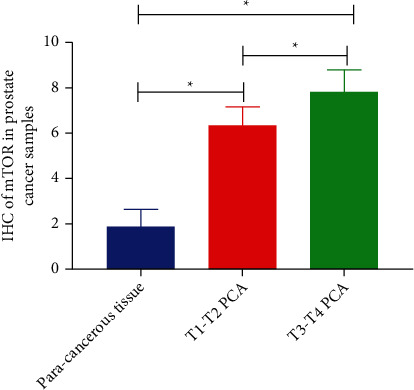
IHC analysis of mTOR in PRAD samples. Compared with paracancerous tissues, the expression of mTOR is upregulated in advanced cancer (*P* < 0.05).

We used in silico tools to investigate the expression of mTOR based on sample types and the race of patients. As shown in Figure 3, the expression of mTOR was upregulated in BLCA and prostate cancer (PRAD) patients (*P* < 0.05, Figures 3(d) and 3(g)). However, the mTOR expression was downregulated in KIRC samples (*P* < 0.05, Figure 3(a)). For KIRC, no obvious difference in overall survival (OS) and disease-free survival (DFS) time was evident between the high and low mTOR expression groups (*P* > 0.05, Figures 3(b) and 3(c)). For BLCA, patients with high expression of mTOR appeared to have shorter DFS time than the low mTOR expression group (*P* < 0.05, Figure 3(e)). No significant difference in OS time was apparent (*P* > 0.05, Figure 3(f)). For PRAD, patients with low expression of mTOR appeared to have shorter DFS time than the high mTOR expression group (*P* < 0.05, Figure 3(h)). No significant difference in OS time was apparent (*P* > 0.05, Figure 3(i)). Regarding mTOR expression in urinary cancer based on race, downregulation was evident in Caucasian, African-American, and Asian KIRC patients (*P* < 0.05, Figure 4(a)). The expression of mTOR was upregulated in Caucasian and African-American BLCA patients (*P* < 0.05), but not in Asian patients (*P* > 0.05, Figure 4(b)). Expression of mTOR was upregulated in Caucasian PRAD patients (*P* < 0.05), but not in African-American patients (*P* > 0.05, Figure 4(c)). Expression profiles of mTOR in Asian PRAD patients could not be acquired from the online database. IHC analysis was used to investigate mTOR expression in 220 pathologically diagnosed PRAD participants voluntarily enrolled from our centers. The feature distribution of PRAD patients has been mentioned in our previous study [50]. Compared with paracancerous tissues, the expression of mTOR was upregulated in advanced PRAD (*P* < 0.05, Figure 5).

**Figure 6:**
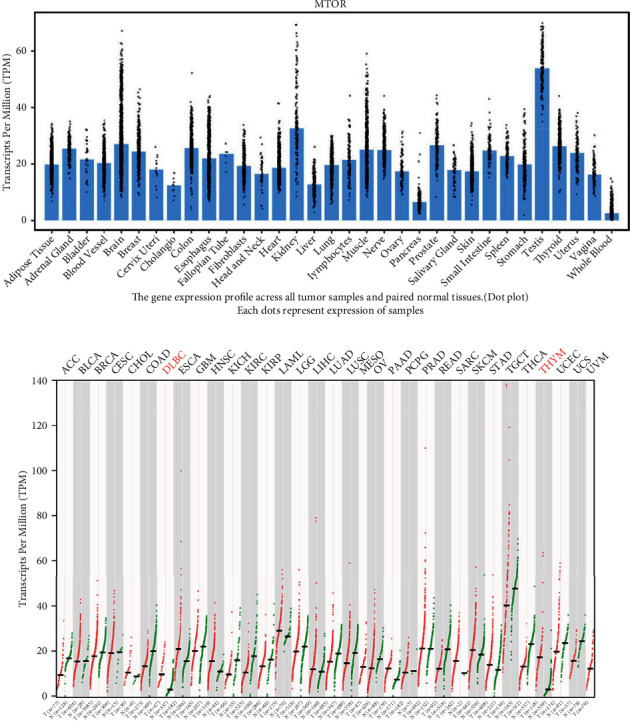
The expression of mTOR in tissues of *Homo sapiens*. Expression of mTOR in various tissues and organs is described in (a). Gene expression profile across all tumor samples and paired normal tissues is shown in (b).

**Figure 7:**
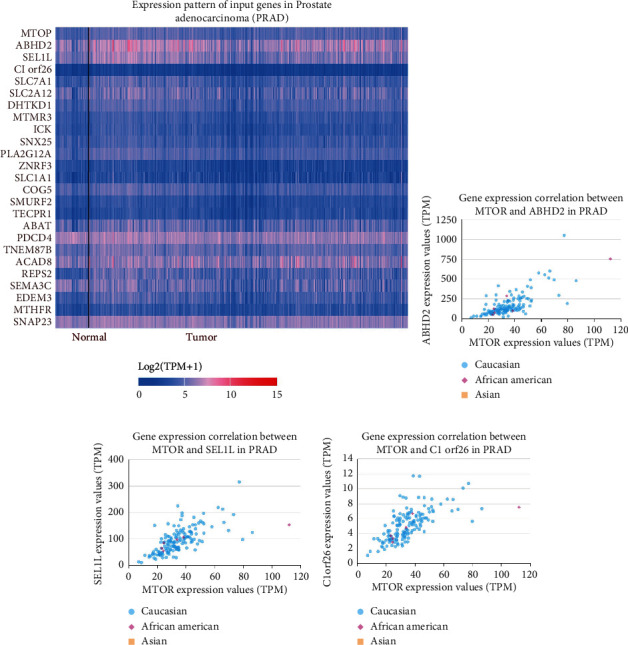
Gene-gene interaction of mTOR in prostate cancer. The expression pattern of input genes in PRAD is shown in (a). Correlation analysis from TCGA samples indicates that ABHD2 (*α*/*β*-hydrolase domain-containing 2 (b)), SEL1L (adaptor subunit of ERAD E3 ubiquitin ligase (c)), and C1ORF26 (SWT1 RNA endoribonuclease homolog (d)) are the most correlated genes with mTOR.

**Figure 8:**
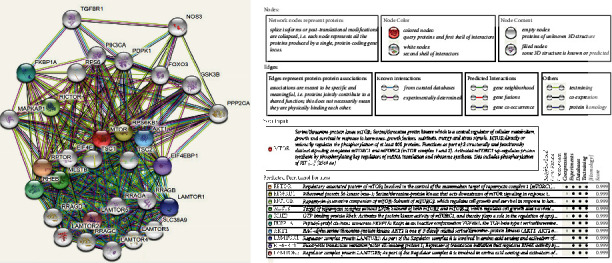
The relationship of mTOR protein assessed by the STRING tools. At least 30 proteins can participate in the interaction with mTOR (a). The most relevant are RPS6KB1 (Ribosomal protein S6 kinase beta-1), LAMTOR5 (Ragulator complex protein), RHEB (GTP-binding protein), MAPKAP1 (Target of rapamycin complex 2 subunit), LAMTOR1 (Ragulator complex protein), RICTOR (Rapamycin-insensitive companion of mTOR), RPTOR (Regulatory-associated protein of mTOR), EIF4EBP1 (Eukaryotic translation initiation factor 4E-binding protein 1), LAMTOR4 (Ragulator complex protein 4), and LAMTOR2 (Ragulator complex protein 2) (b).

**Figure 9:**
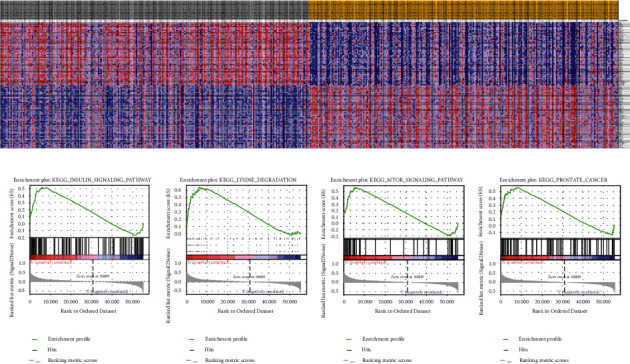
GSEA analysis of samples with high expression of mTOR. Heat map and gene list association profiles are described in (a). GSEA revealed that the insulin signaling pathway (b), lysine degradation pathway (c), and mTOR signaling pathway (d) were enriched in mTOR high-expression group. GSEA also confirmed that mTOR was upregulated in prostate cancer (e).

Furthermore, we adopted an online database to assess the mTOR expression in various tissues and organs of *Homo sapiens*. As described in Figure 6(a), mTOR was highly expressed in organs of the urinary system, especially the kidney and testis. The expression profiles of mTOR differed in different types of tumor tissues (Figure 6(b)). Compared with normal tissues, mTOR expression was downregulated in several types of carcinomas, especially KIRC, testicular tumors, and colon adenocarcinoma. The mTOR expression was especially upregulated in thymoma and lymphoma. In addition, we investigated the gene-gene correlation of mTOR. As shown in Figure 7(a), more than 24 genes interact with the mTOR gene. The most correlated genes contain the following: ABHD2 (*α*/*β*-hydrolase domain-containing 2, Figure 7(b)), SEL1L (adaptor subunit of ERAD E3 ubiquitin ligase, Figure 7(c)), and C1ORF26 (SWT1 RNA endoribonuclease homolog, Figure 7(d)). STRING analysis revealed at least 30 proteins featuring protein-protein crosstalk with mTOR (Figure 8(a)). The most relevant proteins are as follows: RPS6KB1 (Ribosomal protein S6 kinase beta-1), LAMTOR5 (Ragulator complex protein), RHEB (GTP-binding protein), MAPKAP1 (Target of rapamycin complex 2 subunit), LAMTOR1 (Ragulator complex protein), RICTOR (Rapamycin-insensitive companion of mTOR), RPTOR (Regulatory-associated protein of mTOR), EIF4EBP1 (Eukaryotic translation initiation factor 4E-binding protein 1), LAMTOR4 (Ragulator complex protein 4), and LAMTOR2 (Ragulator complex protein 2) (Figure 8(b)). Then Kyoto Encyclopedia of Genes and Genomes (KEGG) functional enrichment was further conducted utilizing gene set enrichment analysis (GSEA). Heat map and gene list association profiles are described in Figure 9(a). GSEA revealed that the insulin signaling pathway (Figure 9(b)), lysine degradation pathway (Figure 9(c)), and mTOR signaling pathway (Figure 9(d)) were enriched in the high mTOR expression group. GSEA also confirmed that mTOR was upregulated in PRAD (Figure 9(e)).

### 3.4. Sensitivity Analysis and Publication Bias

**Figure 10:**
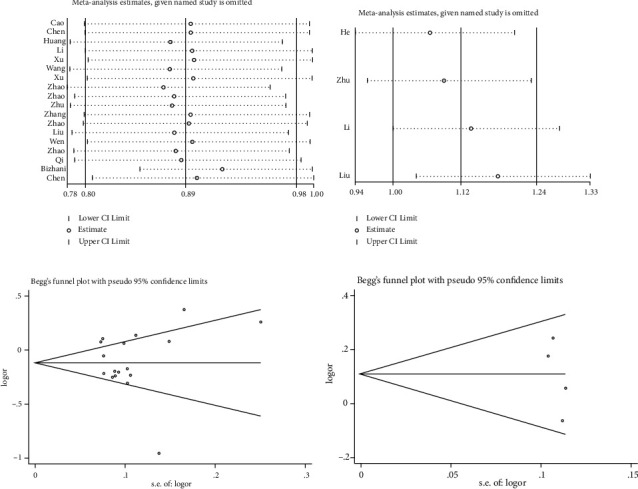
Sensitivity analysis and Begg's funnel plot of mTOR variants. Sensitivity analysis of mTOR variant rs2295080 T/G (a) or rs1883965 G/A (b) indicated that a single study could not influence the significance of ORs. Begg's funnel plot analysis of rs2295080 T/G (c) or rs1883965 G/A (d) polymorphisms under heterozygous comparison model revealed no evidence of publication bias.

Sensitivity analysis was conducted by excluding every single study to evaluate their impact on the overall ORs. As described in Figures 10(a) and 10(b), no single study influenced the significance of ORs for the rs2295080 T/G and rs1883965 G/A mTOR variants (*P* < 0.05). Evaluation of publication bias through Begg's funnel plots did not indicate significant publication bias for all five genetic models of rs2295080 (Figure 10(c), *P* > 0.05) or rs1883965 variants (Figure 10(d), *P* > 0.05).

## 4. Discussion

As a main controller of cell proliferation, mTOR participates in a variety of synthetic metabolic processes including lipogenesis, protein synthesis, and nucleotide biosynthesis. mTOR also inhibits catabolic processes including lysosomal biogenesis and autophagy. Inhibitors of the mTOR signaling pathway have been developed to treat some types of malignant tumors [52, 53]. Previous studies have also linked overexpression or mutation of core genes in the mTOR pathway to the occurrence, invasion, and prognosis of many carcinomas [8, 9]. Genetic variations of mTOR are widespread and could affect the function of protein by altering gene expression.

Several previous publications have assessed the association of mTOR variants rs2295080 T/G or rs1883965 G/A and susceptibility to cancer. However, the sample size of the included studies was insufficient [28, 29]. In 2017, Zhang et al. performed a meta-analysis based on 13 studies and observed a decreased risk of rs2295080 T/G variant on digestive system cancer [54]. However, their conclusion was not confirmed by another pooled analysis [10]. In total, our pooled analysis identified 22 eligible case-control studies comprising 14,747 cancer patients and 16,399 controls on the two mTOR variants. The current study sought to identify all eligible case-control studies to comprehensively assess the association between mTOR variants rs2295080 T/G or rs1883965 G/A and susceptibility to a variety of cancers. For the rs2295080 T/G variation, six studies were on urinary system cancer. For the rs1883965 G/A variation, one study was on urinary cancer. Our analysis does indicate a significant association of mTOR rs2295080 T/G and rs1883965 G/A polymorphism with the risk of cancer.

For the rs2295080 T/G SNP, a stratified analysis by cancer type revealed that the T-allele is a risk factor for urinary and breast cancer. This result is consistent with a recent published meta-analysis [10]. However, the latter study did not identify a significant association between mTOR rs2295080 T/G polymorphism and leukemia susceptibility. The possible reason may be that the sample size was relatively small. Presently, in a subgroup analysis by race, we found that the rs2295080 variation was associated with decreased cancer risk in East Asian populations. In stratification analysis by control source and genotyping method, we observed a significant association of this polymorphism in population-based studies and those using the TaqMan assay. Our results are consistent with the meta-analyses performed by Shao et al. [28]. For the rs1883965 G/A SNP, we observed that individuals carrying A-allele had a 1.12-fold higher likelihood of cancer than those carrying G-allele in the pooled data. Moreover, stratified analysis by cancer type revealed an association of the rs1883965 G/A polymorphism with increased digestive cancer susceptibility in allelic contrast, heterozygous comparison, and dominant model. This finding is consistent with those of Zhu et al. and He et al. [33, 44]. Additionally, we used in silico tools to investigate gene expression profile of mTOR in various types of cancers and normal tissues. mTOR was highly expressed in organs of the urinary system, especially in the kidney and testis tissues. The expression of mTOR was upregulated in BLCA and PRAD patients and was downregulated in KIRC samples. KIRC and BLCA patients displayed no obvious difference in OS between the high and low mTOR expression group. PRAD patients with low expression of mTOR appeared to have shorter DFS time than those with high mTOR expression. As shown in Figure 9, we used GSEA to investigate the possible signaling pathways and cancer correlated with expression of mTOR. We revealed that the mTOR signaling pathway was enriched in high mTOR expression group. Furthermore, the mTOR expression was augmented in PRAD.

Concerning mTOR expression in urinary cancer based on race, downregulated expression was evident in KIRC patients who were Caucasian, African-American, and Asian. For BLCA, the expression of mTOR was upregulated in Caucasians and African-Americans, but not in Asians. For PRAD, mTOR expression was upregulated in Caucasian patients, but not in African-American patients. The expression profiles of mTOR in Asian PRAD patients could not be acquired from the online database. We further used IHC analysis to investigate the mTOR expression in PRAD participants enrolled from our centers. Compared with paracancerous tissues, the expression of mTOR was upregulated in advanced PRAD.

The present study has several limitations. First, according to the inclusion criteria, no case-control study on mTOR rs2295080 T/G and rs1883965 G/A polymorphism was included based on African or Caucasian populations. Further studies on African and Caucasian populations with various tumors are warranted. Second, the sample size of eligible studies for the rs1883965 G/A SNP was insufficient. Studies on many types of cancer including testicular cancer, thyroid carcinoma, thymoma, and lymphoma are very limited. Third, upregulated mTOR expression in advanced PRAD was based on IHS analysis. Further studies are needed to demonstrate whether the mTOR rs2295080 T/G or rs1883965 G/A mutations could affect the expression of mTOR in PRAD. The pathogenesis of cancer is complex, and it is not possible that a single mutation would have a huge impact on the progression. As described in Figure 7, more than 24 genes could participate in interactions with mTOR gene. At least 30 proteins were identified to interact with mTOR (Figure 8). Therefore, gene-gene and gene-environment interactions should be further studied to explore the correlation. Additionally, adjustment analysis of lifestyle or smoking exposure may contribute to better segregation and assessment of different groups. These analyses are warranted to be conducted by future studies.

## 5. Conclusion

In summary, the present study summarized all eligible data for the genetic relationship between the mTOR variants rs2295080 T/G or rs1883965 G/A and susceptibility to different cancers. Our results revealed that rs2295080 T/G polymorphism was associated with susceptibility of urinary cancer, especially in East Asians. The expression of mTOR was upregulated in BLCA and PRAD patients. GSEA revealed that the insulin signaling pathway, lysine degradation pathway, and mTOR signaling pathway were enriched in the high mTOR expression group. The expression of mTOR was positively correlated with tumor malignancy in prostate cancer subjects.

## Figures and Tables

**Table 1:** Study characteristics of mTOR rs2295080 T/G and rs1883965 G/A variants in the current analysis.

Author	Year	Origin	Cancer	Ethnicity	Type	Source of control	Case	Control	Case			Control			HWE	Method
rs2295080 T/G									GG	GT	TT	GG	GT	TT		
Cao	2012	China	KIRC	East Asian	Urinary cancer	Hospital-based	710	760	38	218	454	45	277	438	0.891	TaqMan
Chen	2012	China	PRAD	East Asian	Urinary cancer	Hospital-based	666	708	28	209	429	36	259	413	0.573	TaqMan
Huang	2012	China	ALL	East Asian	Blood cancer	Hospital-based	417	554	23	140	254	21	180	353	0.549	TaqMan
Li	2013	China	PRAD	East Asian	Urinary cancer	Population-based	1004	1051	40	311	653	52	382	617	0.468	TaqMan
Xu	2013	China	GCA	East Asian	Digestive cancer	Hospital-based	753	854	25	246	482	52	305	497	0.569	TaqMan
Wang	2015	China	GCA	East Asian	Digestive cancer	Hospital-based	1002	1003	40	394	568	41	355	607	0.221	TaqMan
Xu	2015	China	CRC	East Asian	Digestive cancer	Hospital-based	737	777	30	225	482	45	273	459	0.602	TaqMan
Zhao	2015	China	ALL	East Asian	Blood cancer	Hospital-based	133	296	15	50	68	12	111	173	<0.001	PCR-RFLP
Zhao	2015	China	AML	East Asian	Blood cancer	Hospital-based	47	296	6	14	27	12	111	173	<0.001	PCR-RFLP
Zhu	2015	China	ESCC	East Asian	Digestive cancer	Population-based	1113	1113	49	390	674	49	362	702	0.788	TaqMan
Zhang	2015	China	KIRC	East Asian	Urinary cancer	Hospital-based	710	760	38	218	454	45	277	438	0.891	TaqMan
Zhao	2016	China	Breast	East Asian	Breast cancer	Hospital-based	560	583	12	197	351	26	212	345	0.358	Sequenom
Liu	2017	China	PRAD	East Asian	Urinary cancer	Hospital-based	413	807	32	145	236	37	316	454	0.052	TaqMan
Wen	2017	China	TCA	East Asian	Others	Population-based	560	500	24	170	366	29	176	295	0.686	TaqMan
Zhao	2017	China	GCA	East Asian	Digestive cancer	Population-based	283	271	15	90	178	11	86	174	0.927	TaqMan
Qi	2017	China	GCA	East Asian	Digestive cancer	Hospital-based	574	912	101	279	194	174	441	297	0.651	TaqMan
Bizhani	2018	Iran	BLCA	West Asian	Urinary cancer	Population-based	235	254	80	90	65	152	76	26	0.001	PCR-RFLP
Chen	2019	China	Breast	East Asian	Breast cancer	Population-based	530	480	19	201	310	37	198	245	0.730	TaqMan

rs1883965 G/A									AA	AG	GG	AA	AG	GG		
He	2013	China	GCA	East Asian	Digestive cancer	Population-based	1125	1196	10	188	927	6	165	1025	0.817	TaqMan
Zhu	2013	China	ESCC	East Asian	Digestive cancer	Population-based	1123	1121	6	209	908	7	174	940	0.732	TaqMan
Li	2013	China	PRAD	East Asian	Urinary cancer	Population-based	1004	1051	8	153	843	7	154	890	0.904	TaqMan
Liu	2014	China	Liver	East Asian	Digestive cancer	Hospital-based	1048	1052	2	165	881	10	160	882	0.365	TaqMan

ALL: acute lymphoblastic leukemia; BLCA: bladder cancer; CRC: colorectal cancer; AML: acute myeloid leukemia; ESCC: esophageal squamous cell carcinoma; GCA: gastric cancer; HWE: Hardy-Weinberg equilibrium of control; PRAD: prostate cancer; TCA: thyroid cancer; KIRC: kidney renal clear cell carcinoma.

**Table 2:** Stratified analysis of mTOR rs2295080 T/G and rs1883965 G/A polymorphisms on cancer susceptibility.

Variables	**N**	Case/control	OR (95% CI)	P_heter_	P	OR (95% CI)	P_heter_	**P**	OR (95% CI)	P_heter_	**P**	OR (95% CI)	P_heter_	**P**	OR (95% CI)	P_heter_	**P**
			M-allele vs. W-allele			MW vs. WW			MM vs. WW			MM+MW vs. WW			MM vs. MW+WW		
rs2295080 T/G																	
Total	18	10447/11979	0.89 (0.80-0.98)	<0.001	0.023	0.88 (0.81-0.96)	0.004	0.004	0.84(0.65-1.07)	<0.001	0.162	0.87 (0.78-0.96)	<0.001	0.008	0.88 (0.70-1.11)	<0.001	0.288
Cancer type																	
Urinary	6	3738/4340	0.76 (0.62-0.94)	<0.001	0.010	0.77 (0.70-0.85)	0.522	<0.001	0.71 (0.44-1.17)	<0.001	0.178	0.74 (0.62-0.88)	0.004	0.001	0.82 (0.53-1.27)	<0.001	0.367
Blood	3	597/1146	1.24 (1.05-1.47)	0.480	0.013	1.06 (0.85-1.33)	0.691	0.578	2.12 (1.36-3.30)	0.263	0.001	1.17 (0.95-1.44)	0.722	0.142	2.08 (1.34-3.22)	0.225	0.001
Digestive	6	4462/4930	0.95 (0.83-1.08)	0.003	0.443	0.98 (0.85-1.13)	0.026	0.773	0.84 (0.71-1.01)	0.108	0.058	0.96 (0.82-1.12)	0.006	0.598	0.85 (0.72-1.01)	0.241	0.067
Breast	2	1090/1063	0.79 (0.68-0.91)	0.364	0.001	0.86 (0.72-1.03)	0.475	0.093	0.42 (0.27-0.66)	0.810	<0.001	0.80 (0.68-0.95)	0.380	0.012	0.45 (0.29-0.71)	0.909	<0.001
Others	1	560/500	0.79 (0.64-0.97)	-	0.027	0.78 (0.60-1.01)	-	0.060	0.67 (0.38-1.17)	-	0.158	0.76 (0.59-0.98)	-	0.033	0.73 (0.42-1.27)	-	0.261
Ethnicity																	
East Asian	17	10212/11725	0.92 (0.85-1.00)	<0.001	0.044	0.89 (0.82-0.97)	0.012	0.006	0.90 (0.72-1.11)	<0.001	0.315	0.89 (0.81-0.98)	0.001	0.013	0.93 (0.76-1.14)	<0.001	0.492
West Asian	1	235/254	0.38 (0.29-0.50)	-	<0.001	0.47 (0.27-0.82)	-	0.008	0.21 (0.12-0.36)	-	<0.001	0.30 (0.18-0.49)	-	<0.001	0.35 (0.24-0.50)	-	<0.001
Source																	
HB	12	6722/8310	0.94 (0.85-1.03)	<0.001	0.194	0.89 (0.81-0.98)	0.041	0.022	0.97 (0.74-1.27)	<0.001	0.803	0.90 (0.81-1.00)	0.006	0.056	1.00 (0.78-1.30)	<0.001	0.976
PB	6	3725/3669	0.78 (0.60-1.00)	<0.001	0.048	0.84 (0.69-1.02)	0.007	0.077	0.61 (0.37-1.02)	<0.001	0.061	0.77 (0.60-0.99)	<0.001	0.043	0.68 (0.45-1.02)	0.001	0.065
Method																	
TaqMan	14	9472/10550	0.90 (0.83-0.98)	<0.001	0.011	0.88 (0.81-0.97)	0.005	0.008	0.84 (0.70-1.01)	0.011	0.066	0.88 (0.80-0.97)	0.001	0.008	0.88 (0.74-1.04)	0.036	0.134
PCR-RFLP	3	415/846	0.89 (0.34-2.30)	<0.001	0.807	0.77 (0.44-1.34)	0.047	0.360	1.25 (0.16-9.53)	<0.001	0.829	0.75 (0.29-1.96)	<0.001	0.555	1.47 (0.26-8.18)	<0.001	0.659
Sequenom	1	560/583	0.84 (0.69-1.03)	-	0.089	0.91 (0.72-1.17)	-	0.467	0.45 (0.23-0.91)	-	0.027	0.86 (0.68-1.09)	-	0.225	0.47 (0.23-0.94)	-	0.033
rs1883965 G/A																	
Total	4	4300/4420	1.12 (1.00-1.24)	0.203	0.045	1.15 (1.02-1.29)	0.484	0.019	0.91 (0.54-1.54)	0.114	0.733	1.14 (1.02-1.27)	0.328	0.026	0.89 (0.53-1.51)	0.120	0.673
Cancer type																	
Urinary	1	1004/1051	1.06 (0.85-1.32)	-	0.621	1.05 (0.82-1.34)	-	0.700	1.21 (0.44-3.34)	-	0.718	1.06 (0.83-1.34)	-	0.655	1.20 (0.43-3.32)	-	0.728
Digestive	3	3296/3369	1.13 (1.00-1.28)	0.115	0.044	1.18 (1.03-1.34)	0.415	0.014	0.82 (0.44-1.53)	0.056	0.538	1.16 (1.02-1.32)	0.228	0.022	0.80 (0.43-1.49)	0.061	0.483
Source																	
HB	1	1048/1052	0.94 (0.75-1.17)	-	0.563	1.03 (0.81-1.31)	-	0.792	0.20 (0.04-0.92)	-	0.038	0.98 (0.78-1.24)	-	0.889	0.20 (0.04-0.91)	-	0.038
PB	3	3252/3368	1.18 (1.04-1.33)	0.484	0.009	1.19 (1.04-1.36)	0.489	0.011	1.28 (0.71-2.32)	0.626	0.416	1.19 (1.05-1.36)	0.479	0.009	1.25 (0.69-2.26)	0.627	0.467

HB: hospital based; PCR-RFLP: polymerase chain reaction-restriction fragment length polymorphism; PB: population based. ^a^Number of case-control studies. *P*_heter_: *P* value of heterogeneity test.

## Data Availability

All the data generated in the present research are available by the corresponding authors after reasonable request.

## Abbreviations

mTOR: Mammalian target of rapamycin
ORs: Odds ratios
GSEA: Gene set enrichment analysis
IHS: Immunohistochemical staining
ABHD2: *α*/*β*-Hydrolase domain-containing 2
CIs: Confidence intervals
KIRC: Kidney renal clear cell carcinoma
RHEB: GTP-binding protein
BLCA: Bladder cancer
SNP: Single-nucleotide polymorphism
PRAD: Prostate cancer
SEL1L: Adaptor subunit of ERAD E3 ubiquitin ligase
PCR-RFLP: Polymerase chain reaction-restriction fragment length polymorphism
C1ORF26: RNA endoribonuclease homolog
DFS: Disease-free survival
HWE: Hardy-Weinberg equilibrium
RPS6KB1: Ribosomal protein S6 kinase beta-1
OS: Overall survival.
